# Comparison of the Serum Metabolic Fingerprint of Different Exercise Modes in Men with and without Metabolic Syndrome

**DOI:** 10.3390/metabo9060116

**Published:** 2019-06-15

**Authors:** Aikaterina Siopi, Olga Deda, Vasiliki Manou, Ioannis Kosmidis, Despina Komninou, Nikolaos Raikos, Georgios A. Theodoridis, Vassilis Mougios

**Affiliations:** 1 School of Physical Education and Sport Science, Aristotle University of Thessaloniki, 54124 Thessaloniki, Greece; vmanou@phed.auth.gr (V.M.); ikosmidia@phed.auth.gr (I.K.); mougios@auth.gr (V.M.); 2 School of Chemistry, Aristotle University of Thessaloniki, 54124 Thessaloniki, Greece; oliadmy@gmail.com (O.D.); gtheodor@chem.auth.gr (G.A.T.); 3 Center for Interdisciplinary Research and Innovation, BIOMIC_AUTh (CIRI-AUTH), Aristotle University of Thessaloniki, 57001 Thessaloniki, Greece; raikos@med.auth.gr; 4 FoodOmicsGR RI, Center for Interdisciplinary Research and Innovation, AUTh node (CIRI-AUTH), Aristotle University of Thessaloniki, 57001 Thessaloniki, Greece; 5 Department of Nutrition and Dietetics, Alexander Technological Educational Institute of Thessaloniki, 57400 Sindos, Greece; komninoud@gmail.com; 6 Laboratory of Forensic Medicine and Toxicology, School of Medicine, Aristotle University of Thessaloniki, 54124 Thessaloniki, Greece

**Keywords:** metabolomics, serum metabolites, metabolic syndrome, exercise mode

## Abstract

Exercise plays a beneficial role in the treatment of metabolic syndrome (MetS). Metabolomics can provide new insights and facilitate the optimization of exercise prescription. This study aimed to investigate whether the response of the human serum metabolic fingerprint to exercise depends on exercise mode or the presence of MetS. Twenty-three sedentary men (nine with MetS and fourteen healthy) completed four trials: Resting, high-intensity interval exercise (HIIE), continuous moderate-intensity exercise (CME), and resistance exercise (RE). Blood samples were collected pre-exercise, immediately after exercise, and 1 h post-exercise for targeted metabolomic analysis in serum by liquid chromatography–mass spectrometry. Time exerted the strongest differentiating effect, followed by exercise mode. The largest changes from baseline were found in the immediate post-exercise samples. RE caused the strongest responses overall, followed by HIIE, while CME had minimal effect. Unlike previous results in urine, no valid model could separate the two groups in serum. Exercise exerted a beneficial effect on prominent serum biomarkers of metabolic risks, such as branched-chain amino acids, alanine, acetylcarnitine, choline, and betaine. These findings contribute to the ongoing research efforts to map the molecular responses to exercise and to optimize exercise guidelines for individuals at cardiometabolic risk.

## 1. Introduction

The metabolic syndrome (MetS), a condition that involves cardiometabolic risk factors, such as visceral obesity, hyperglycemia, dyslipidemia, and hypertension, shows alarmingly increasing rates [1,2]. MetS increases the risk of type 2 diabetes, cardiovascular disease, and all-cause mortality [1,3,4]. Although the pathophysiological mechanisms behind the manifestation of MetS are far from clear, insulin resistance appears as the most widely accepted and unifying cause, as it affects many major metabolic pathways [5]. Recent advances in the field of metabolomics have helped broaden the characterization of the metabolic profile of obesity-related diseases. Metabolites, such as branched-chain amino acids (BCAA), aromatic amino acids, glutamine, glutamate, glycine, alanine, serine, proline, lysine, histidine, α-hydroxybutyrate, kynurenate, acylcarnitines, lysophosphatidylcholines, nicotinuric acid, trimethylamine-*Ν*-oxide, choline, and betaine, have all been connected to obesity, insulin resistance, MetS, diabetes, non-alcoholic fatty liver disease (NAFLD) and cardiovascular disease [6,7,8,9,10,11,12,13,14,15,16,17]. 

Exercise plays an important role in the prevention and treatment of MetS, as it exerts a beneficial effect on the associated risks [18,19,20]. It takes a single bout of exercise to acutely improve the lipidemic profile, blood pressure, insulin sensitivity, glucose homeostasis, as well as immunological, vascular, and hemostatic functions [21]. This means that the cardiometabolic benefits of exercise training could be greatly attributed to recent exercise. The majority of relevant studies have implemented continuous moderate-intensity exercise (CME). Studies that have implemented different exercise modes, i.e., high-intensity interval exercise (HIIE) and resistance exercise (RE), have also reported improvements in insulin sensitivity and MetS risk factors [19,22,23].

Moreover, they show that different exercise modes or their combination could have better effects on insulin resistance than CME [24,25,26]. Different exercise modes possibly exert their beneficial effects through different molecular mechanisms [19,27]. These mechanisms and the best exercise mode (or the best combination of exercise modes) for optimal clinical outcomes in individuals with MetS are yet to be determined.

The approach of metabolomics can provide new insights into exercise metabolism and help deepen our understanding of the beneficial effects of exercise [28,29,30]. As far as we know, the acute effects of the aforementioned exercise modes have not been directly compared using metabolomics. Moreover, the vast majority of exercise metabolomic studies so far have been on competitive athletes or healthy, young, and active individuals. There are limited data from exercise metabolomic studies on individuals with cardiometabolic risk factors or diseases. Most of these studies investigated the effects of exercise training [31,32,33,34,35,36,37,38], a few investigated the effects of acute exercise [39,40,41], and all included sedentary, overweight or obese individuals with metabolic risk factors, diabetes, or NAFLD, but not MetS.

Thus, this study focuses on investigating whether the serum metabolic fingerprint responds differently depending on the presence of MetS or the mode of exercise. More specifically, we compared the effects of a resting trial (REST) along with three fundamentally different exercise modes (HIIE, CME, and RE) on the serum metabolic fingerprints of sedentary, middle-aged men with or without MetS. Moreover, we compared the results in serum with previously published results from the same study in urine [42]. 

## 2. Results

Characteristics of the participants have been previously described [42]. Briefly, there is a significant difference between the two groups (i.e., MetS and Healthy) in terms of the metabolic syndrome, visceral fat, maximal oxygen uptake, and in the homeostatic model assessment 2 for insulin resistance. Five serum metabolites, namely, alanine, glutamate, homocysteine, norvaline–valine, and proline, were significantly higher in MetS in comparison to Healthy at baseline. Serum leucine–isoleucine was also numerically higher in MetS, but the difference was marginally significant (*p* = 0.051). On the other hand, nicotinamide and spermine were lower in MetS compared to Healthy at baseline. 

As previously described [42], there were no significant differences in total distance covered between the HIIE and CME trials or in exercise intensity between groups. The change in plasma volume did not differ significantly among exercise trials or groups (post- to pre-exercise plasma volume ratios, HIIE, 0.97 ± 0.09 and 0.96 ± 0.03; CME, 1.00 ± 0.07 and 1.01 ± 0.05; RE, 0.95 ± 0.08 and 0.96 ± 0.06 for MetS and Healthy, respectively). Total daily energy intake, macronutrient intake, and step count did not differ significantly between groups or trials, with the exception that protein intake on the day before the CME trial was higher than on the day before the REST trial. However, since total energy, carbohydrate, and fat intake did not differ significantly between those days, this finding was not considered important during the interpretation of the results. 

The serum glucose concentration presented significant trial × time interaction, as well as significant main effects of time and group, with MetS being higher than Healthy (Supplementary Table S1). In HIIE, glucose was significantly higher immediately post-exercise (1 h time point) compared to pre-exercise (0 h time point) and 1 h post-exercise (2 h time point). In RE, glucose was significantly lower at 2 h compared to 0 and 1 h. Immediately after CME, glucose was significantly lower compared to HIIE and RE. The serum insulin concentration presented significant trial × time interaction, as well as significant main effects of trial and time (Supplementary Table S1). It is noteworthy that insulin had a large increase immediately after RE, compared to the other trials, and then returned to baseline at 2 h. There was no significant interaction involving the group factor, which suggests than changes in glucose or insulin exhibited similar trends in both groups.

The spectrophotometrically determined serum lactate concentration was significantly different between trials immediately after exercise (Supplementary Table S2). Lactate increased significantly after RE and HIIE, but not after CME. The largest increase was after RE. There were no significant differences between groups. Lactate measured spectrophotometrically was significantly correlated with lactate determined by UPLC-MS/MS analysis (r = 0.885, *p* < 0.001).

### 2.1. Univariate Metabolomic Analysis

**Figure 1:**
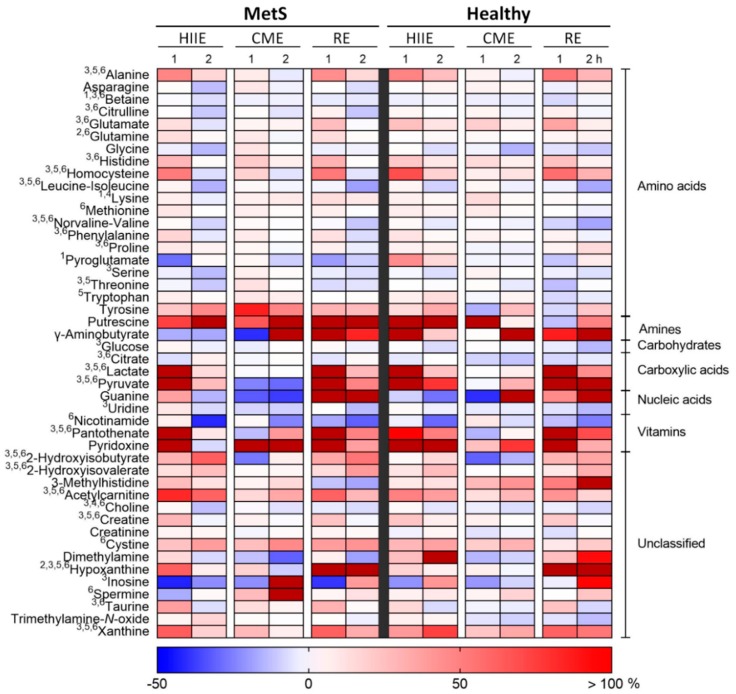
Percentage change from baseline in serum metabolites at 1 and 2 h for the three exercise modes (HIIE,high-intensity interval exercise; CME, continuous moderate-intensity exercise; and RE,resistance exercise) in each group (MetS,metabolic syndrome; and Healthy). Significant interactions (*p* < 0.05) from the three-way repeated–measure ANOVA are noted as follows: ^1^Exercise mode × time × group, ^2^time × group, ^3^exercise mode × time. Notation for the significant main effects: ^4^group, ^5^exercise mode, ^6^time.

The heat map in Figure 1 presents the results of the three-way analysis of variance (ANOVA) on the serum metabolite levels determined through UPLC-MS/MS analysis. Four metabolites, namely, betaine, hypoxanthine, lysine, and pyroglutamate, presented a significant three-way interaction. In particular, after immediately decreasing following RE in both groups, betaine (precisely, glycine betaine, or trimethylglycine) remained low 1 h after RE in MetS, while it increased in Healthy. Betaine also showed a delayed decrease after HIIE (2 h) in MetS, whereas in Healthy the decrease appeared at 1 h and remained through 2 h. Hypoxanthine, which presented a large increase immediately after RE, started decreasing 1 h later in MetS, while remaining increased in Healthy. Lysine exhibited the highest increase immediately after CME in Healthy and less so after HIIE, while it did not change remarkably in MetS. Pyroglutamate deceased immediately after HIIE in MetS, while it increased in Healthy. 

There was no significant two-way interaction of exercise mode and group. Only glutamine and hypoxanthine showed a significant time × group interaction. These metabolites were significantly higher in MetS at 1 h compared to the other two time points. Glutamine did not change in Healthy, while hypoxanthine was higher at 1 and 2 h, compared to 0 h. Twenty-seven serum metabolites, namely, 2-hydroxyisobutyrate, 2-hydroxyisovalerate, acetylcarnitine, alanine, betaine, choline, citrate, citrulline, creatine, glucose, glutamate, histidine, homocysteine, hypoxanthine, inosine, lactate, leucine-isoleucine, norvaline-valine, pantothenate, phenylalanine, proline, pyruvate, serine, taurine, threonine, uridine, and xanthine, showed significant exercise mode × time interactions. This was due to differences between 1 h and the other two time points; and between CME and the other two exercise modes. 

**Figure 2:**
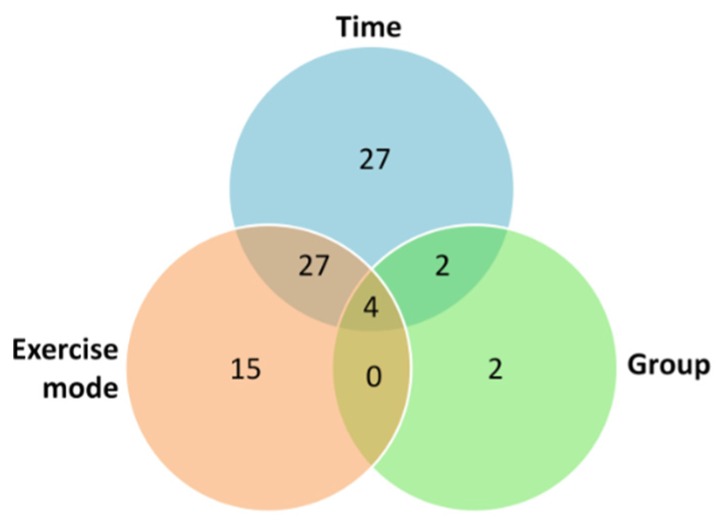
The results of three-way ANOVA in a Venn diagram. Numbers represent the sums of significant main effects and interactions with regards to all 46 serum metabolites determined by UPLC-MS/MS in serum (*p* < 0.05).

Regarding the main effects, choline and lysine were significantly different between groups. Fifteen metabolites were found significantly different among exercise modes. Twelve of them differed significantly between HIIE and CME; nine differed between CME and RE; and, six differed between HIIE and RE. There was a significant change over time in twenty-seven metabolites. This time effect was located in the difference of the first post-exercise sample (1 h) from the other samples. Moreover, twelve metabolites were significantly different between 0 and 2 h. The Venn diagram presented in Figure 2 summarizes the significant main effects and interactions with regards to all 46 serum metabolites. The *p* values and effect size values for all significant changes are summarized in Supplementary Table S4.

### 2.2. Multivariate Metabolomic Analysis

**Figure 3:**
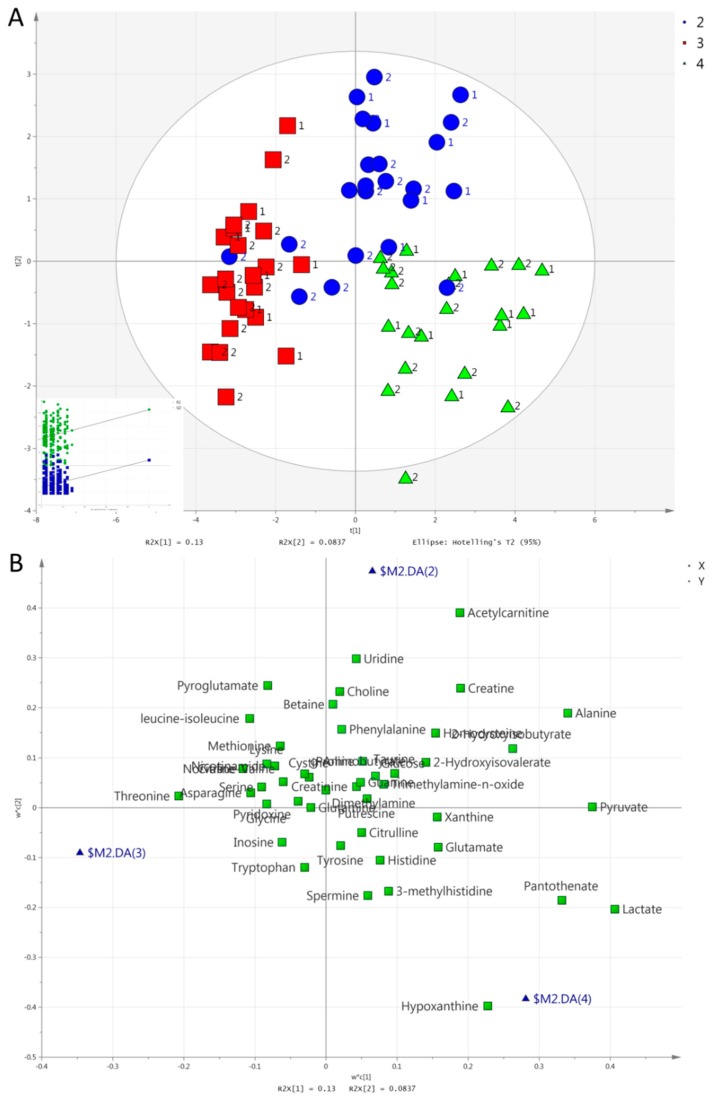
(**A**). Score plots concerning serum samples for the a partial least square discriminant analysis (PLS-DA) model comparing exercise modes at 1 h: HIIE (blue circles), CME (red squares), RE (green triangles). Inserts (subfigure) are permutation plots. The number 1 is used for the MetS group and 2 for the Healthy group. (**B**). Loading plot for the respective PLS-DA model. Blue triangles represent HIIE (2), CME (3), and RE (4).

**Figure 4:**
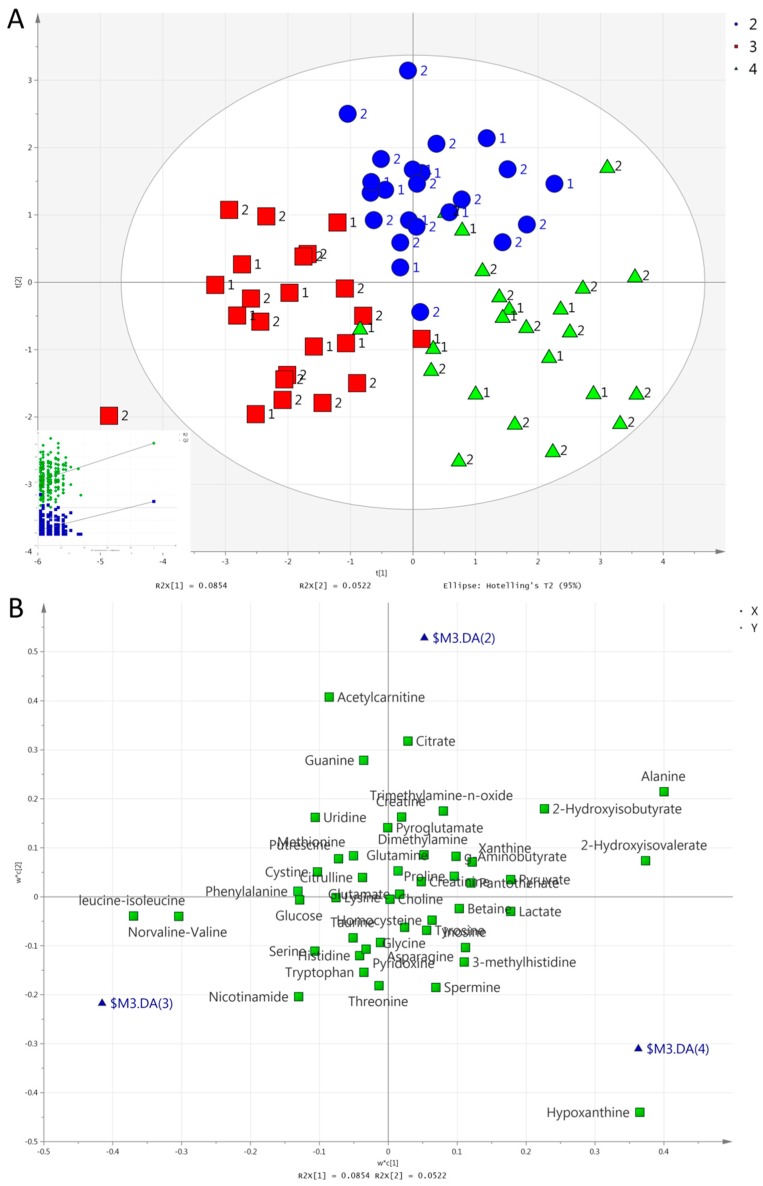
(**A**). Score plot concerning serum samples for the PLS-DA model comparing exercise modes at 2 h: HIIE (blue circles), CME (red squares), RE (green triangles). Inserts (subfigure) are permutation plots. The number 1 is used for the MetS group and 2 for the Healthy group. (**B**). Loading plot for the respective PLS-DA model. Blue triangles represent HIIE (2), CME (3), and RE (4).

We consecutively set group, exercise mode, and time as the Y variable to perform a partial least square discriminant analysis (PLS-DA). Unlike in urine [42], no valid model could separate the two groups in serum. In contrast to the results from urine, however, PLS-DA separated all three exercise modes in one model in serum. Figure 3 and Figure 4 show the score and loading plots for the separation of the exercise modes at both post-exercise time points (i.e., 1 h and 2 h). Pairwise PLS-DA was also performed, and discrimination by exercise mode was achieved at 1 h between HIIE and CME; between CME and RE; and between HIIE and RE (Supplementary Figure S1). There was also a significant difference at 2 h between HIIE and CME; and between CME and RE. Between HIIE and RE, the model was marginally significant (CV-ANOVA, *p* = 0.070; Supplementary Figure S2).

**Figure 5:**
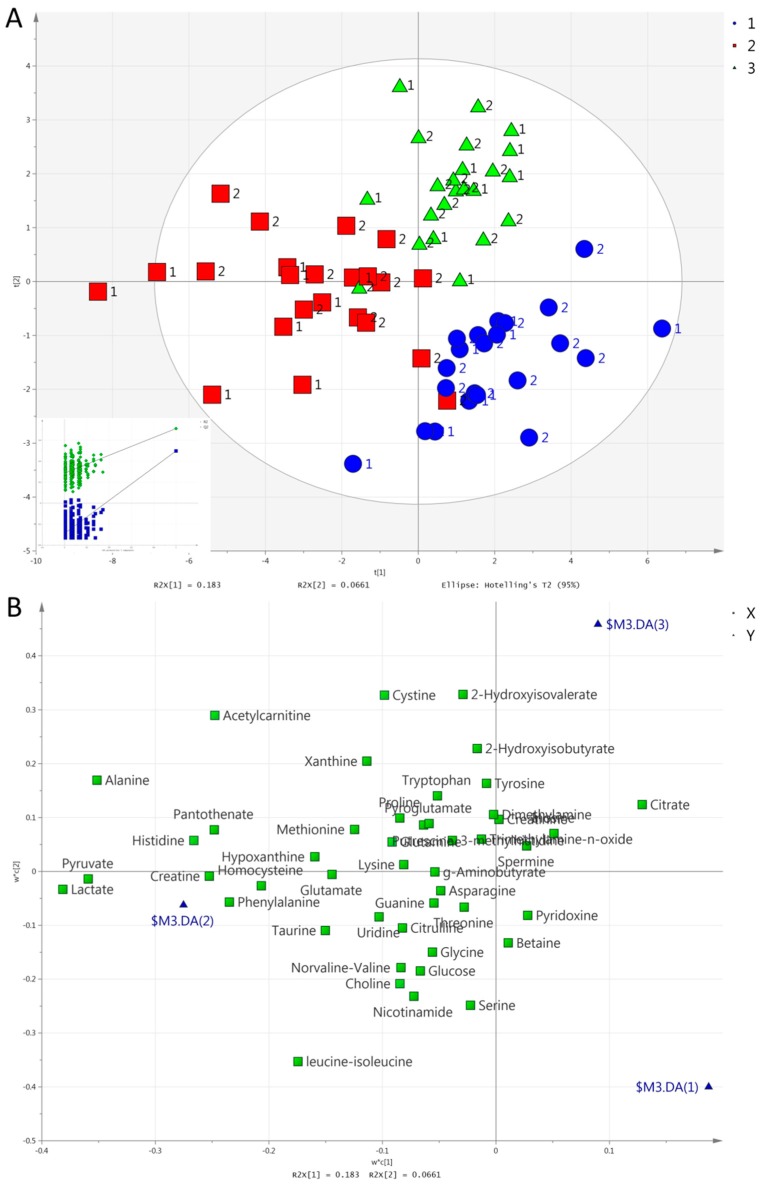
(**A**). Score plot concerning serum samples for the PLS-DA model for HIIE: 0 h (blue circles), 1 h (red squares), 2 h (green triangles). Inserts (subfigure) are permutation plots. The number 1 is used for the MetS group and 2 for the Healthy group. (**B**). Loading plot for the respective PLS-DA model. Blue triangles represent 0 h (1), 1 h (2), and 2 h (3).

**Figure 6:**
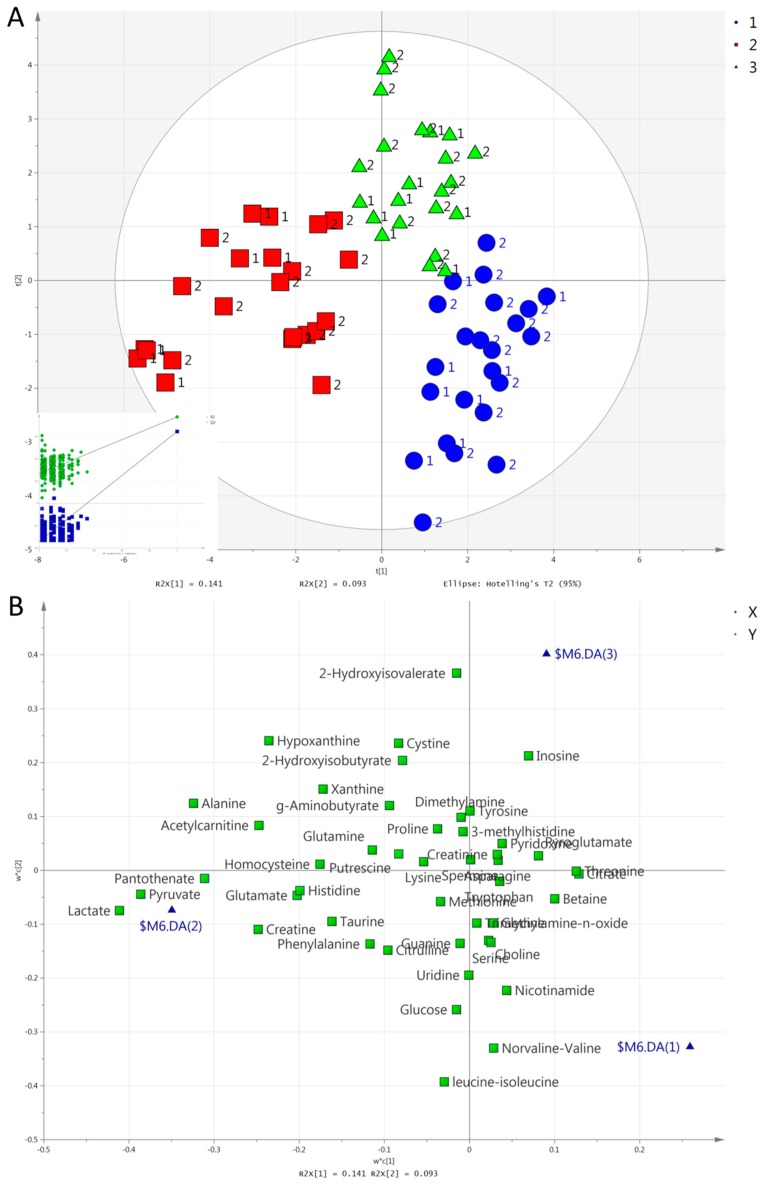
(**A**). Score plot concerning serum samples for the PLS-DA model for RE: 0 h (blue circles), 1 h (red squares), 2 h (green triangles). Inserts (subfigure) are permutation plots. The number 1 is used for the MetS group and 2 for the Healthy group. (**B**). Loading plot for the respective PLS-DA model. Blue triangles represent 0 h (1), 1 h (2), and 2 h (3).

Valid models also separated all three time points for HIIE and RE (Figure 5 and Figure 6, respectively), but not for CME. Pairwise separation by time was achieved for HIIE between 0 and 1 h; 0 and 2 h; and 1 and 2 h (Supplementary Figure S3). Like in urine [42], no valid models were constructed for CME. All time points were significantly separated in RE. The Supplementary Figure S4 presents the score plots for the pairwise comparisons of the different time points in RE.

Supplementary Table S3 shows a summary of the characteristics of all valid models concerning serum samples. The important serum metabolites to explain the differences for valid PLS-DA models of each pairwise comparison, according to their variable importance on projection (VIP) scores, are shown in Table 1.

The metabolites responsible for the separation of all three exercise modes or time points (in order of descending VIP scores) are as follows:

(i) Concerning the separation of exercise modes at 1 h (Figure 3), HIIE caused a larger increase in acetylcarnitine compared to the other exercises. RE caused a larger increase in lactate, pyruvate, hypoxanthine, and pantothenate compared to the other exercises. Both HIIE and RE caused similar increases in alanine, 2-hydroxyisobutyrate, and creatine, while CME caused a lesser increase in alanine, a decrease in 2-hydroxyisobutyrate, and no change in creatine. CME increased threonine, while HIIE and RE decreased it;
(ii) Concerning the separation of exercise modes at 2 h (Figure 4), HIIE caused a higher increase in acetylcarnitine in comparison to other exercises. RE caused a higher increase in hypoxanthine in comparison to other exercises. Both HIIE and RE caused similar increases in alanine, 2-hydroxyisovalerate, and 2-hydroxyisobutyrate. CME caused no change in alanine or 2-hydroxyisobutyrate, but it caused a decrease in 2-hydroxyisobutyrate. CME caused a lesser decrease in leucine-isoleucine and norvaline-valine than HIIE or RE did;
(iii) The separation of time points in HIIE (Figure 5) was mainly due to the larger increases in lactate, pyruvate, alanine, acetylcarnitine, histidine, pantothenate, and phenylalanine in the first post-exercise sample (1 h). In addition, leucine-isoleucine, while remaining unchanged at 1 h, presented a decrease at 2 h;
(iv) The separation of time points in RE (Figure 6) was mainly due to the larger increases in lactate, pyruvate, alanine, hypoxanthine, pantothenate, creatine, and acetylcarnitine at 1 h. In addition, 2-hydroxyisovalerate presented a greater increase at 2 h than at 1 h.

## 3. Discussion

This study investigated the alterations in the human serum metabolic fingerprint, due to the effect of three independent factors, that is, exercise mode, time after exercise, and the presence or not of MetS. A targeted UPLC-MS/MS analytical method, which identifies polar compounds (e.g., amino acids, amines, sugars, organic acids, nucleic acid components, and water-soluble vitamins), was used. As far as we know, this was the first time that the serum metabolic fingerprints of three fundamentally different exercise modes were compared. Following both univariate (ANOVA) and multivariate (PLS-DA) statistical analysis, we showed that time had the strongest differentiating effect on the metabolic fingerprints, with exercise mode coming second. The largest changes were seen in the immediate post-exercise samples. Of the three exercise modes, the greatest responses were due to RE, while CME had minimal effect. These results are in line with previously reported results from the same study in urine samples [42]. Unlike in urine, however, where we found evidence for decreased metabolic flexibility of MetS in response to exercise, no valid model could separate the two groups in serum. 

### 3.1. Between-Group Comparison of Baseline Metabolic Fingerprints

In blood, increases in metabolites, such as leucine, isoleucine, glucose, urate, and acetylcarnitine, and decreases in glycine have been associated with impaired fasting glucose regulation [43,44]. Moreover, decreased glycine and increased valine, phenylalanine, and combined glutamine and glutamate have been associated with insulin resistance [45]. Phenylalanine, leucine-isoleucine, valine, tyrosine, methionine, alanine, and histidine have been found to distinguish metabolically well from metabolically compromised individuals independent of body mass index (BMI) [6]. Serum BCAA were also higher in metabolically unhealthy, centrally obese patients compared to metabolically healthy, peripherally obese individuals [46]. In a similar male Mediterranean group with MetS, a plasma amino acid pattern mainly involving BCAA and aromatic amino acids was positively associated with MetS, while a second pattern involving glutamine, glycine, serine, and asparagine was inversely associated with MetS [16]. In the present study, individuals with MetS had higher baseline values of serum BCAA, alanine, glutamate, homocysteine, and proline, in agreement with the literature [17]. However, it should be noted that most large observational studies were on individuals with obesity, cardiometabolic risk factors and/or type 2 diabetes, who were compared to healthy, lean individuals. By contrast, in the present study we compared individuals with MetS to individuals without MetS, but with similar BMI and body composition [42], except for visceral adiposity, in order to dissect the effects of central obesity in particular. This could explain the lack of significant differences in baseline values of other aforementioned biomarkers and the lack of separation of the baseline metabolic fingerprints of the two groups after multivariate statistical analysis. 

### 3.2. Between-Group Comparison of Post-Exercise Metabolic Fingerprints—Serum vs. Urine

In contrast to urine [42], there was no separation of the post-exercise serum metabolic fingerprints between groups. With the particular UPLC-MS/MS analytical method, we monitor the levels of 64 metabolites in urine and 46 in serum. Overall, 23 urinary metabolites were not detected in serum, while five serum metabolites were not detected in urine. For example, kynurenate, riboflavin, and thymine, three out of the ten metabolites that were responsible for the discrimination of the post-exercise urinary metabolic fingerprints between groups [42], were not part of the serum metabolic fingerprint. These differences are expected and are usually attributed to differences in consistency between biological matrices. Compared to serum, urine is a less complex aqueous solution; therefore, it is easier to identify polar compounds in it. Moreover, the concentration of a metabolite may differ in the two matrices by orders of magnitude; therefore, it may be detected in one, but fail to reach the limit of detection in the other. Lastly, ion suppression, which is the loss of a compound’s signal because of co-elution and ionization of a similar, interfering compound, can be very different in different biological matrices. Many researchers view urine as the ideal source of biomarkers, as it is under no homeostatic control, non-invasively accessible, more stable, and less complex than other biofluids [47,48]. Indeed, urine provided evidence for a blunted metabolic response to exercise in MetS individuals [42], while serum did not in the present study. However, this does not cancel the utility of blood sampling. In fact, the separation of the metabolic fingerprints of different exercise modes or different time points was notably stronger in serum compared to urine, as will be discussed below. 

### 3.3. Comparison of the Metabolic Fingerprints of Different Exercise Modes

With regards to comparing the three exercise modes used in this study, it is clear that RE had the greatest effect on the serum metabolic fingerprint, at least, as shown using this particular method, which mainly identifies polar compounds. HIIE had a lesser effect, and CME had the weakest impact. These results are similar to published results from the same study, where RE was the most effective of the three exercise modes in increasing the circulating levels of irisin, an exercise myokine, immediately after exercise [49]. 

In serum, results were similar to urine [42], with an even stronger separation of exercise modes at all time points. In addition (and in contrast to urine), it was possible to separate the metabolic fingerprints of all exercise modes in a single model, both immediately and 1 h after exercising. The reason for that could be that any changes in metabolite levels are naturally manifested in blood first and in urine later. Therefore, in blood we can detect the immediate responses to exercise, while in urine the more delayed ones, after metabolites have passed from blood to urine and homeostatic/anaplerotic mechanisms have pushed blood metabolites to baseline levels. Perhaps urine sampling at any time between the first post-exercise urine sample (1 h after exercise), and the second one (3 h after exercise) employed in our previous study [42] could have given an equally strong separation to that observed in serum 1 h after exercise. That may be of importance for when designing future exercise metabolomic studies.

The three exercise modes chosen for this study design utilize the full spectrum of energy systems: From the aerobic system, which predominates during CME, to the lactate system, which has a considerable contribution during HIIE, to the ATP-phosphocreatine system, which, along with the lactate system, apparently predominate during the exercise bouts in RE [50]. The separation of the fingerprints of the three exercise modes in this study at both post-exercise time points reflects these differences in energy system contribution. For example, HIIE caused a larger increase in acetylcarnitine compared to the other exercises, while RE caused a larger increase in lactate, pyruvate, hypoxanthine, and pantothenate. During short, intense, recurrent efforts, such as in the RE and HIIE trials, there is a high degradation of AMP in skeletal muscle, producing inosine monophosphate, inosine and hypoxanthine in sequence. The conversion of pyruvate to alanine through the activity of alanine aminotransferase can explain the larger increase in alanine after RE and HIIE compared to CME. These results are generally in accordance with our findings in urine [42] and with studies by other investigators [51,52,53]. Another study that investigated the effects of RE on the serum metabolome of 10 young healthy males reported significant increases in metabolites like 2-hydroxybutyrate, 2-oxoisocaproate, 3-hydroxyisobutyrate, alanine, hypoxanthine, lactate, pyruvate, and succinate, as well as significant decreases in isoleucine, leucine, lysine, ornithine, and valine [54].

### 3.4. The Response of Biomarkers of Metabolic Risk

As previously mentioned, several metabolomic studies have associated certain metabolites (mostly in blood) with obesity, insulin resistance and MetS-related disorders. In the present study, exercise had a significant effect on some of the most prominent among those biomarkers. In serum, BCAA, leucine-isoleucine and norvaline-valine, decreased after HIIE and RE but not after CME. At the same time, there was an increase in valine’s catabolic product, 2-hydroxyisovalerate. Therefore, HIIE and RE, but not CME of the particular duration and intensity, can be prescribed for the reduction of increased BCAA levels, which are strongly associated with insulin resistance. On the other hand, CME was the only exercise to cause a decrease in serum alanine, which is also positively associated with MetS-related disorders. Acetylcarnitine, an index of energy substrate oxidation, increased immediately after both HIIE and RE, but remained increased only after HIIE. Exercise also decreased the gut microbiota-related biomarkers of cardiometabolic risks, choline and betaine. These examples demonstrate how exercise metabolomics could be used to tailor exercise parameters, such as exercise mode and intensity, to achieve personalized goals for cardiometabolic risk reduction. 

We were not able to collect expiratory gases during exercise to measure energy expenditure through indirect calorimetry and substrate oxidation rates through the respiratory exchange ratio. This is the main limitation of the study, as measuring these parameters would have facilitated a more precise matching of the two mostly aerobic exercise trials and a better correlation of metabolite changes with carbohydrate and lipid utilization. Instead, we matched the three exercise trials based on exercise duration and, in the case of CME and HIIE, total workload (defined as total distance covered), which is more applicable to everyday life. Another limitation of our study was that exercise intensity was matched in relative terms (that is, percentage of maximal heart rate) between groups, which resulted in lower absolute intensities for MetS, since MetS had lower maximal aerobic capacity than Healthy. This should be taken into account when interpreting some of the results. 

## 4. Materials and Methods

Materials and methods for this study have been previously described [42]. Briefly, twenty-three sedentary men were divided into two groups: MetS (n = 9) and Healthy (n = 14). Participants were assigned to the MetS group if they met at least three out of the five criteria for the metabolic syndrome [55]. All participants provided written informed consent before entering the study, which was approved by the institutional ethics committee (protocol number of the approval 858/16.1.13) and was conducted in accordance with the Helsinki declaration of 1975, as revised in 2008.

Using a crossover design, all volunteers participated in four trials: REST (which served as the control trial), HIIE, CME, and RE, as previously described [42]. After providing baseline blood samples, participants either performed exercise in the HIIE, CME, and RE trials or remained seated and relaxed in the REST trial. The three exercise trials were timed to end one hour after the baseline sample collection. Blood samples were collected in each exercise trial at baseline (set as 0 h), immediately after exercising (1 h), and 1 h post-exercise (2 h). Blood samples were also collected at the same time points in the REST trial. 

Blood samples (8 mL) were left to clot, and then kept refrigerated, pending transport to the laboratory, where they were centrifuged at 3000× *g* at 4 °C for 10 min. Four aliquots (~1 mL each) of the obtained serum were stored at −80 °C until analysis. In addition, in the exercise trials, 2 mL of blood was collected in EDTA tubes at baseline and immediately after exercise for hemoglobin, and hematocrit determination to calculate plasma volume changes due to exercise. Hemoglobin was analyzed spectrophotometrically using a commercially available kit (Spinreact, Santa Coloma, Spain). Hematocrit was determined by the microhematocrit method [56]. Plasma volume change was calculated based on the equation of Dill and Costill [57]. In a subgroup of five MetS and eleven Healthy participants, plasma volume change was compared among exercise trials with a 2 (group) × 3 (trial) ANOVA with repeated measures on trial. 

Serum samples at baseline and immediately after exercise were analyzed spectrophotometrically for lactate as described [28]. Those data were analyzed with a 2 (group) × 3 (trial) × 2 (time) ANOVA with repeated measures on trial and time using SPSS, v. 22 (IBM, Chicago, IL). Effect sizes (ES) were calculated as partial eta-squared. Serum samples were also assayed for glucose spectrophotometrically using a commercially available kit (Spinreact, Santa Coloma, Spain), and insulin by enzyme immunoassay, using a kit from IBL International (Hamburg, Germany). Serum glucose and insulin concentrations were analyzed with a 2 (group) × 4 (trial) × 3 (time) ANOVA with repeated measures on trial and time. 

All serum samples were analyzed through a targeted hydrophilic-interaction ultraperformance liquid chromatography–tandem mass spectrometry (UPLC-MS/MS) method, as previously described [42,58]. Serum samples were thawed and mixed just prior to processing; 200 μL was diluted with 600 μL of cold acetonitrile-methanol 1:1 (v/v), mixed for 20 min, and centrifuged at 15,000× *g* for 36 min. As an external validation of the UPLC-MS/MS analysis, lactate levels determined by UPLC-MS/MS were correlated with those determined spectrophotometrically using Spearman’s correlation.

For each serum metabolite measured through UPLC-MS/MS, the mean of the values at 0 h in the four trials was used to compare baseline levels between groups. For the metabolomic analysis, the peak area of each metabolite in the serum samples of the exercise trials was normalized to the respective value in the REST trial to correct for batch effect and to isolate the effects of exercise from other confounding factors, such as feeding, diurnal variation, and possible glomerular hyperfiltration associated with obesity or pre-diabetes [59]. For the univariate analysis, a 2 (group) × 3 (exercise mode) × 3 (time) ANOVA with repeated measures on exercise mode and time was performed on the 46 monitored metabolites in serum. Significant main effects of exercise mode and time were followed up with post-hoc tests using Sidak’s adjustment for multiple comparisons, whereas significant interactions were followed up with simple main effects analysis using Sidak’s adjustment too. Multivariate statistical analysis was performed using SIMCA 13.0 (Umetrics, Umea, Sweden) as previously described [42]. Group, exercise mode, and time were each set as the Y variable for PLS-DA of all possible pairwise comparisons. 

## 5. Conclusions

We have monitored the changes of 46 serum metabolites after acute bouts of three exercise modes in men with and without MetS, using a UPLC-MS/MS-based metabolomics approach. The differences found between exercise modes reflect differences in the predominant energy systems. The strongest response was seen after RE, followed by HIIE, while CME’s effect was weak. Unlike in urine, where we have previously reported a diminished response to exercise in individuals with MetS, no valid model could separate the two groups in serum. However, exercise exerted a beneficial effect on prominent serum biomarkers of metabolic risks, such as BCAA, alanine, acetylcarnitine, choline, and betaine. These results add to the ongoing research efforts to map the molecular responses to exercise and to optimize exercise guidelines for individuals at cardiometabolic risk.

## Figures and Tables

**Table 1:** Important serum metabolites that explain the valid PLS-DA models.

	Exercise Mode						Time					
	1 h			2 h			HIIE			RE		
	HIIE vs. CME	HIIE vs. RE	CME vs. RE	HIIE vs. CME	HIIE vs. RE	CME vs. RE	0 vs. 1 h	0 vs. 2 h	1 vs. 2 h	0 vs. 1 h	0 vs. 2 h	1 vs. 2 h
2−Hydroxyisobutyrate	−0.42 ***	-	0.75 ***	-	-	-	-	-	-	-	-	-
2−Hydroxyisovalerate	−0.14 *	-	-	-	-	0.38 ***	-	0.26 ***	-	-	0.35 ***	0.24 ***
Acetylcarnitine	−0.31 ***	-	-	-	−0.21 ***	-	0.60 ***	0.46 ***	-	0.49 ***	-	-
Alanine	−0.28 ***	-	0.43 ***	−0.20 ***	-	0.28 ***	0.48 ***	0.22 ***	−0.18 ***	0.50 ***	0.23 ***	−0.18 ***
Choline	−0.10 *	-	-	-	-	-	-	-	-	-	-	-
Creatine	−0.18 ***	-	0.19 **	-	-	-	0.26 ***	-	-	0.22 ***	-	−0.20 ***
Cystine	-	-	-	-	-	-	-	0.37 ***	-	-	-	-
Histidine	-	-	-	-	-	-	0.28 ***	-	-	-	-	-
Homocysteine	−0.30 *	-	0.42 **	-	-	-	-	-	-	-	-	-
Hypoxanthine	-	1.12 ***	1.47 ***	-	1.32 ***	1.29 ***	-	-	-	1.68 ***	1.32 ***	-
Lactate	−0.61 ***	0.60 ***	3.04 ***	-	-	-	1.54 ***	-	−0.52 ***	3.29 ***	0.37 ***	−0.67 ***
Leucine−isoleucine	-	-	-	0.12 ***	-	−0.16 ***	-	−0.11 ***	−0.14 ***	-	−0.18 ***	−0.15 ***
Norvaline−valine	-	-	-	-	-	-	-	-	-	-	−0.15 ***	-
Pantothenate	−0.65 ***	0.71 ***	3.75 ***	-	-	-	1.38 ***	-	-	3.01 ***	-	−0.59 ***
Phenylalanine	-	-	-	-	-	-	0.15 ***	-	−0.11 ***	-	-	-
Pyruvate	−0.80 ***	0.37 *	5.91 ***	-	-	-	3.61 ***	-	−0.67 ***	6.29 ***	0.99 ***	−0.73 ***
Threonine	-	-	−0.15 ***	-	-	-	-	-	-	-	-	-
Uridine	−0.12 *	-	-	-	-	-	-	-	-	-	-	-

Numbers indicate fold change and appear wherever a metabolite contributed to the discrimination. For example, the first number, −0.42, means that the value in CME was 0.42 fold lower than the value in HIIE. * *p* < 0.05, ** *p* < 0.01, *** *p* < 0.001, significant difference following Student’s *t* test.

## Supplementary Materials

The following are available online at https://www.mdpi.com/2218-1989/9/6/116/s1, Table S1: Serum glucose and insulin concentrations. Table S2: Serum lactate concentration. Table S3: Summary of model characteristics from PLS-DA multivariate analysis, concerning serum samples. Table S4: Summary of *p* values and effect sizes (ES) for all significant effects from the univariate statistical analysis. Figure S1: Score plots for the valid PLS-DA models of pairwise comparisons of exercise modes at 1 h. Figure S2: Score plots for the valid PLS-DA models of pairwise comparisons of exercise modes at 2 h. Figure S3: Score plots for the valid PLS-DA models of pairwise comparisons of different time points for HIIE. Figure S4: Score plots for the valid PLS-DA models of pairwise comparisons of different time points for RE.
