# Supplementary material for: Comparison of the Serum Metabolic Fingerprint of Different Exercise Modes in Men with and without Metabolic Syndrome
Source: Metabolites. 2019 Jun 15;9(6):116. doi: 10.3390/metabo9060116 (PMC6631338; doi:10.3390/metabo9060116)
Supplement: Supplementary file 1 [file metabolites-09-00116-s001.pdf]

Supplementary Table S1. Serum glucose and insulin concentrations

Glucose (mg/dL)

	MetS			Healthy		
	0 h	1 h	2 h	0 h	1 h	2 h
REST	91 ± 12	90 ± 12	91 ± 10	73 ± 10	74 ± 10	76 ± 10
HIIE	89 ± 10	97 ± 14[*‡]	88 ± 9	74 ± 5	79 ± 10[*‡]	75 ± 6
CME	85 ± 9	84 ± 10	85 ± 7	75 ± 6	75 ± 8	77 ± 8
RE	87 ± 6	95 ± 6[‡]	83 ± 7[*]	77 ± 6	79 ± 10[‡]	71 ± 7[*]

Insulin (mU/L)

	MetS			Healthy		
	0 h	1 h	2 h	0 h	1 h	2 h
REST	22 ± 7	22 ± 9	20 ± 9	18 ± 5	15 ± 6	16 ± 6
HIIE	23 ± 11	25 ± 11	19 ± 12[*]	19 ± 6	18 ± 9	11 ± 5[*]
CME	22 ± 7	18 ± 8	17 ± 7[*#]	18 ± 6	18 ± 8	12 ± 7[*#]
RE	20 ± 9[†]	39 ± 14[*#†‡]	22 ± 10[†]	15 ± 7[†]	29 ± 15[*#†‡]	15 ± 6[†]

Data are mean ± SD. [*]Significantly different from the other time points. [#]Significantly different from the resting trial (REST). [†]Significantly different from high-intensity interval exercise (HIIE). [‡]Significantly different from continuous moderate-intensity exercise (CME). $p < 0.05$, based on simple main effects analysis following the significant time x trial interactions that resulted from three-way ANOVA. RE, resistance exercise; MetS, metabolic syndrome.

Supplementary Table S2. Serum lactate concentration (mmol/L)

	MetS		Healthy	
	0 h	1 h	0 h	1 h
HIIE	2.6 ± 0.5	8.0 ± 1.7$^{*\#}$	2.8 ± 0.9	7.3 ± 2.6$^{*\#}$
CME	3.2 ± 0.8	2.9 ± 0.6$^{\#}$	2.5 ± 0.6	2.4 ± 0.8$^{\#}$
RE	2.8 ± 0.5	12.6 ± 2.3$^{*\#}$	2.7 ± 1.0	13.1 ± 3.5$^{*\#}$

Data are mean ± SD. *Significantly different from 0 h. $^{\#}$Significantly different from other trials. $p < 0.05$, based on simple main effects analysis following significant time x trial interactions that resulted from three-way ANOVA.

Supplementary Table S3. Summary of model characteristics from partial least square discriminant analysis (PLS-DA) multivariate analysis, concerning serum samples.

Comparisons		Predictive Components	R^2X	R^2Y	Q^2Y	CV-ANOVA, *p* value
1 h	All trials	2	0.213	0.608	0.369	2.44E-08
	HIIE vs. CME	2	0.298	0.777	0.647	9.82E-09
	HIIE vs. RE	2	0.135	0.823	0.286	4.20E-03
	CME vs. RE	2	0.277	0.902	0.768	1.14E-12
2 h	All trials	2	0.138	0.603	0.304	1.67E-06
	HIIE vs. CME	2	0.190	0.877	0.518	2.82E-06
	CME vs. RE	2	0.146	0.867	0.541	2.85E-05
HIIE	All time point	3	0.324	0.734	0.528	8.50E-17
	0 vs. 1 h	2	0.315	0.804	0.667	5.08E-09
	0 vs. 2 h	2	0.213	0.908	0.747	8.04E-12
	1 vs. 2 h	2	0.239	0.828	0.573	7.89E-07
RE	All times point	3	0.367	0.783	0.676	3.43E-27
	0 vs. 1 h	2	0.352	0.878	0.790	1.37E-13
	0 vs. 2 h	2	0.285	0.843	0.660	2.84E-09
	1 vs. 2 h	2	0.273	0.864	0.732	7.01E-11

CV-ANOVA: analysis of variance of cross-validated predictive residuals.

Supplementary Table S4. Summary of P values and effect sizes (ES) for all significant effects from the univariate statistical analysis (Figure 1).

		**P value**	**ES**
**Exercise mode x time x group interaction**	Betaine	0.024	0.133
	Hypoxanthine	0.039	0.112
	Lysine	0.049	0.106
	Pyroglutamate	0.049	0.117
**Time x group interaction**	Glutamine	0.026	0.160
	Hypoxanthine	0.036	0.147
**Exercise mode x time interaction**	2-Hydroxyisobutyrate	0.000	0.381
	2-Hydroxyisovalerate	0.000	0.504
	Acetylcarnitine	0.000	0.600
	Alanine	0.000	0.617
	Betaine	0.001	0.118
	Choline	0.003	0.170
	Citrate	0.000	0.268
	Citrulline	0.029	0.119
	Creatine	0.000	0.269
	Glucose	0.003	0.174
	Glutamate	0.001	0.204
	Histidine	0.007	0.154
	Homocysteine	0.037	0.136
	Hypoxanthine	0.000	0.634
	Inosine	0.026	0.122
	Lactate	0.000	0.867
	Leucine-Isoleucine	0.000	0.477
	Norvaline-Valine	0.001	0.260
	Pantothenate	0.000	0.556
	Phenylalanine	0.000	0.216
	Proline	0.000	0.221
	Pyruvate	0.000	0.745
	Serine	0.002	0.185

	Taurine	0.000	0.220
	Threonine	0.000	0.311
	Uridine	0.001	0.191
	Xanthine	0.016	0.122
**Main effects: group**	Choline	0.038	0.190
	Lysine	0.039	0.180
**Main effects: exercise mode**	2-Hydroxyisobutyrate	0.000	0.389
	2-Hydroxyisovalerate	0.000	0.403
	Acetylcarnitine	0.000	0.395
	Alanine	0.000	0.643
	Creatine	0.011	0.119
	Homocysteine	0.021	0.194
	Hypoxanthine	0.000	0.670
	Lactate	0.000	0.867
	Leucine-Isoleucine	0.002	0.251
	Norvaline-Valine	0.037	0.145
	Pantothenate	0.000	0.622
	Pyruvate	0.000	0.796
	Threonine	0.034	0.148
	Tryptophan	0.039	0.143
	Xanthine	0.000	0.308
**Main effects: time**	2-Hydroxyisobutyrate	0.016	0.180
	2-Hydroxyisovalerate	0.000	0.522
	Acetylcarnitine	0.000	0.745
	Alanine	0.000	0.805
	Betaine	0.042	0.155
	Choline	0.012	0.189
	Citrate	0.011	0.192
	Citrulline	0.008	0.232
	Creatine	0.000	0.550
	Cystine	0.000	0.626
	Glutamate	0.000	0.325

	Glutamine	0.005	0.226
	Histidine	0.000	0.512
	Homocysteine	0.002	0.265
	Hypoxanthine	0.000	0.502
	Lactate	0.000	0.907
	Leucine-Isoleucine	0.000	0.677
	Methionine	0.050	0.133
	Nicotinamide	0.002	0.251
	Norvaline-Valine	0.012	0.226
	Pantothenate	0.000	0.515
	Phenylalanine	0.000	0.439
	Proline	0.020	0.171
	Pyruvate	0.000	0.785
	Spermine	0.004	0.316
	Taurine	0.003	0.236
	Xanthine	0.002	0.307

Results from three-way ANOVA. Effect sizes (ES) were calculated as partial eta-squared.

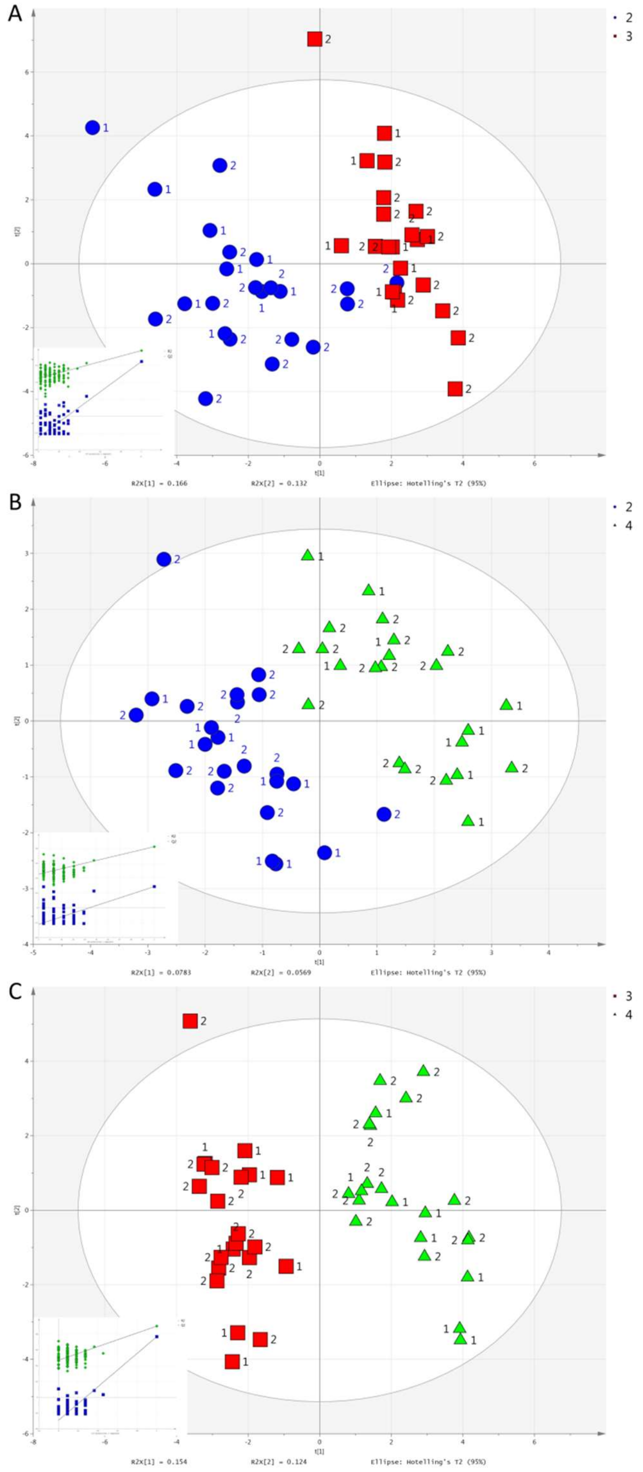

Supplementary Figure S1. Score plots concerning serum samples for the PLS-DA models of pairwise comparisons of exercise modes at 1 h: (**A**) HIIE (blue circles) vs. CME (red squares); (**B**) CME vs. RE (green triangles); (**C**) HIIE vs. RE. Inserts are permutation plots. The MetS group is represented as 1 and the Healthy group as 2.

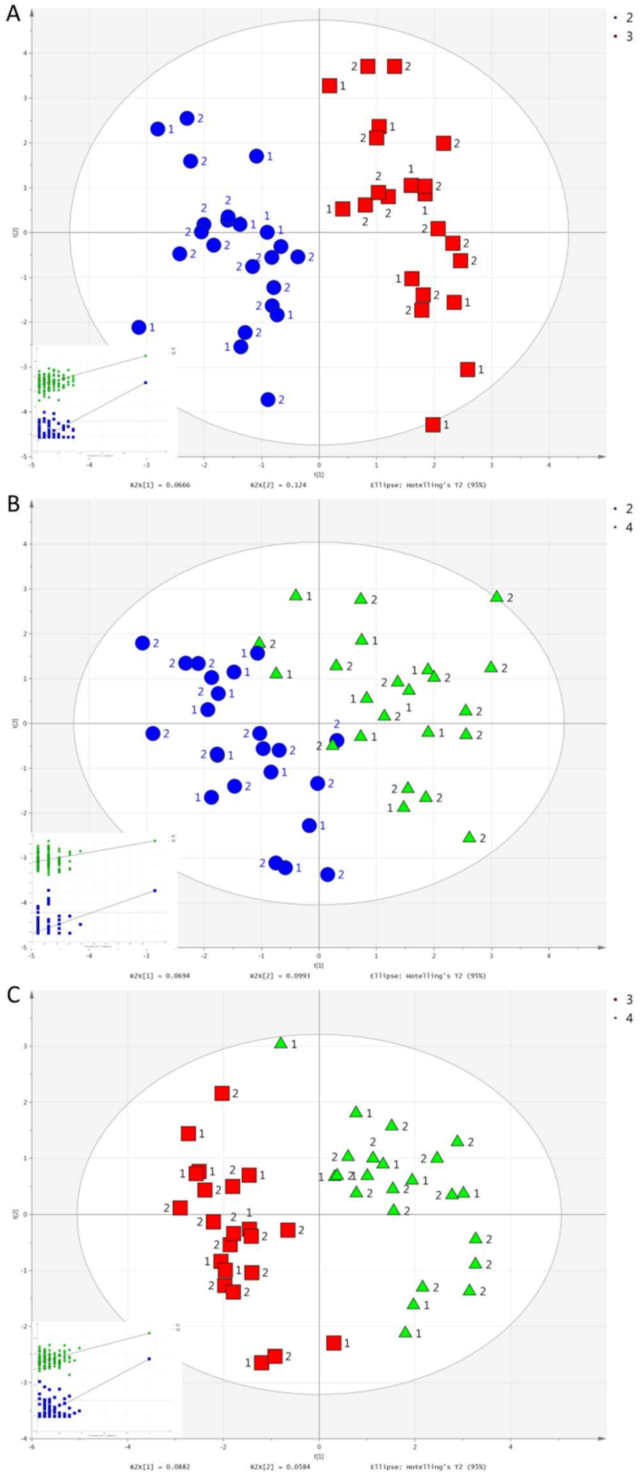

Supplementary Figure S2. Score plots concerning serum samples for the PLS-DA models of pairwise comparisons of exercise modes at 2 h: (**A**) HIIE (blue circles) vs. CME (red squares); (**B**) CME vs. RE (green triangles); (**C**) HIIE vs. RE. Inserts are permutation plots. The MetS group is represented as 1 and the Healthy group as 2.

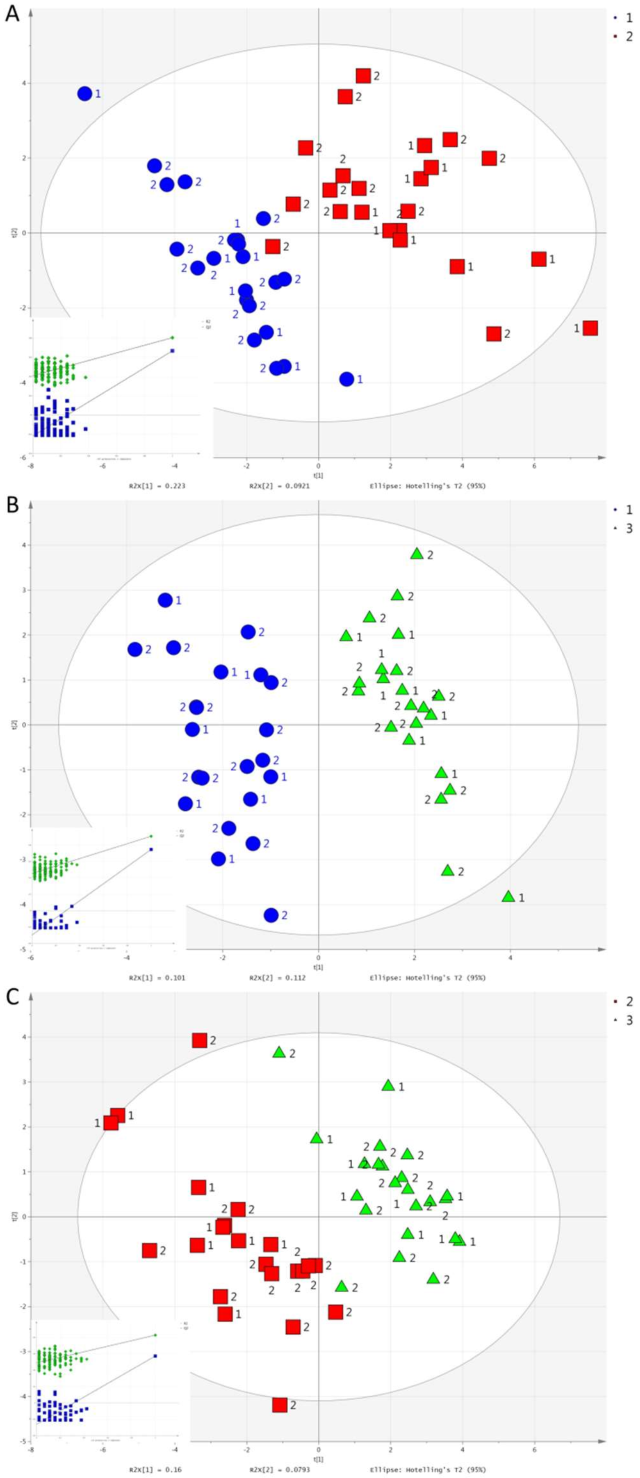

Supplementary Figure S3. Score plots concerning serum samples for the PLS-DA models of pairwise comparisons for HIIE: (**A**) 0 h (blue circles) vs. 1 h (red squares); (**B**) 0 h vs. 2 h (green triangles); (**C**) 1 h vs. 2 h. Inserts are permutation plots. The MetS group is represented as 1 and the Healthy group as 2.

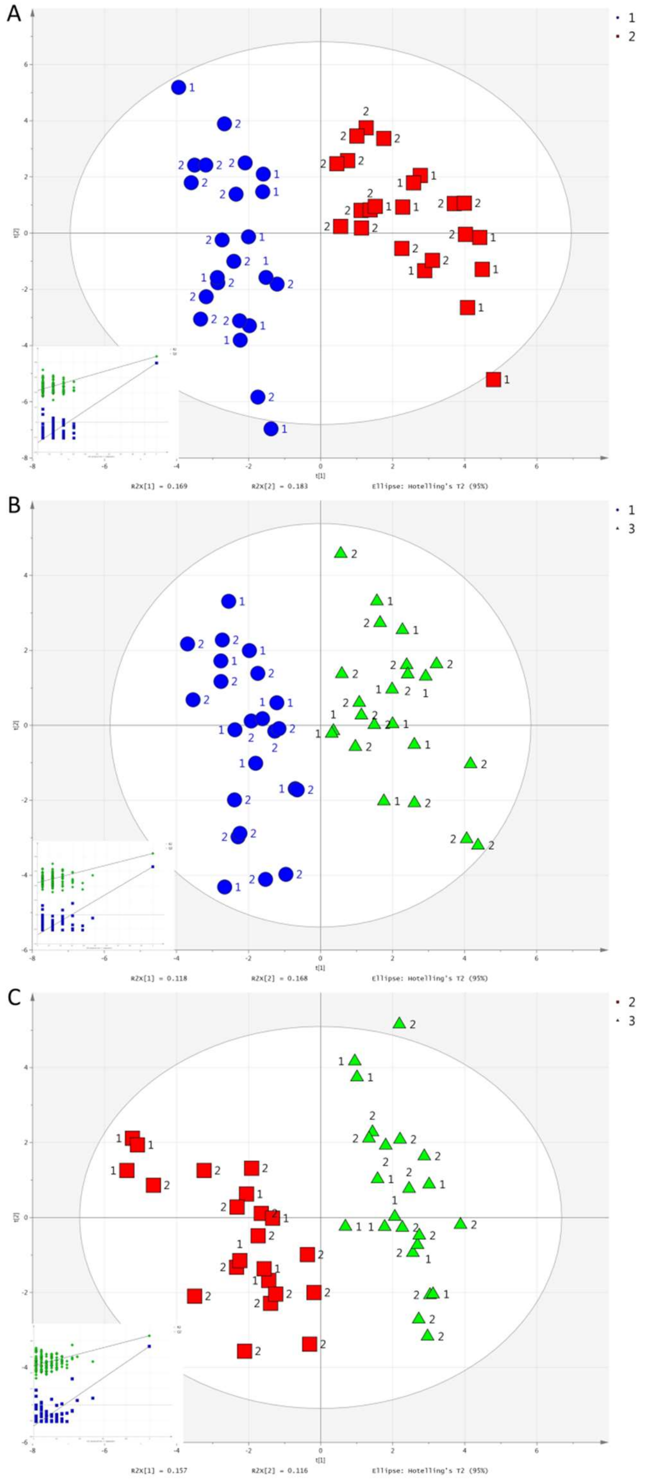

Supplementary Figure S4. Score plots concerning serum samples for the PLS-DA models of pairwise comparisons for RE: (**A**) 0 h (blue circles) vs. 1 h (red squares); (**B**) 0 h vs. 2 h (green triangles); (**C**) 1 h vs. 2 h. Inserts are permutation plots. The MetS group is represented as 1 and the Healthy group as 2.
